# Real-life use of tolvaptan in ADPKD: a retrospective analysis of a large Canadian cohort

**DOI:** 10.1038/s41598-023-48638-9

**Published:** 2023-12-14

**Authors:** Luca Calvaruso, Kevin Yau, Pedram Akbari, Fatemah Nasri, Saima Khowaja, Bill Wang, Amirreza Haghighi, Korosh Khalili, York Pei

**Affiliations:** 1 Division of Nephrology, Department of Medicine, University Health Network, Toronto, ON Canada; 2 University of Toronto, Toronto, ON Canada; 3 U.O.C. Nefrologia, Fondazione Policlinico Universitario A. Gemelli, IRCCS, Rome, Italy; 4 Department of Medical Imaging, University Health Network and University of Toronto, Toronto, ON Canada; 5 Chair, Patient Liaison Advisory Group of the International Society of Nephrology, Hong Kong, China; 6 Division of Genetics, Brigham and Women’s Hospital and Harvard Medical School, Boston, MA USA

**Keywords:** Medical research, Translational research

## Abstract

Tolvaptan is the first disease-modifying drug proven to slow eGFR decline in high-risk patients with ADPKD. However, barriers from the patient perspective to its use in real-life settings have not been systemically examined in a large cohort. This was a single-center, retrospective study of 523 existing or new patients with ADPKD followed at the Center for Innovative Management of PKD in Toronto, Ontario, between January 1, 2016 to December 31, 2018. All patients underwent clinical assessment including total kidney volume measurements and Mayo Clinic Imaging Class (MCIC). Those who were deemed to be at high risk were offered tolvaptan with their preference (yes or no) and reasons for their choices recorded. Overall, 315/523 (60%) patients had MCIC 1C-1E; however, only 96 (30%) of them were treated with tolvaptan at their last follow-up. Among these high-risk patients, those not treated versus treated with tolvaptan were more likely to have a higher eGFR (82 ± 26 vs. 61 ± 27 ml/min/1.73 m^2^), CKD stages 1–2 (79% vs. 41%), and MCIC 1C (63% vs. 31%). The most common reasons provided for not taking tolvaptan were lifestyle preference related to the aquaretic effect (51%), older age ≥ 60 (12%), and pregnancy/family planning (6%). In this real-world experience, at least 60% of patients with ADPKD considered to be at high risk for progression to ESKD by imaging were not treated with tolvaptan; most of them had early stages of CKD with well-preserved eGFR and as such, were prime targets for tolvaptan therapy to slow disease progression. Given that the most common reason for tolvaptan refusal was the concern for intolerability of the aquaretic side-effect, strategies to mitigate this may help to reduce this barrier to tolvaptan therapy.

## Introduction

Autosomal dominant polycystic kidney disease (ADPKD) is the most common monogenic hereditary kidney disease with a life-time risk of approximately 1:1000^1^ and is the fourth leading cause of end-stage kidney disease (ESKD) in developed countries, accounting for 5–10% of patients with ESKD^2,3^. It is characterized by the progressive development of renal cysts leading to renal dysfunction and multiple extra-renal complications^4,5^. Mutations of two genes, *PKD1* and *PKD2*, account for most of the genetically resolved cases^6–8^. Comprehensive *PKD1* mutation screening from recent studies has further refined genotype–phenotype correlation and confirmed a strong correlation between mutation class and kidney disease severity: on average, *PKD1* protein-truncating (PT) mutations (i.e. frameshift, nonsense, and canonical splice site mutations, and large deletions) and *PKD1* inframe insertions/deletions (indels, IF) are associated with the most severe disease, followed by intermediate disease in *PKD1* non-truncating (NT) mutations (i.e. missense variants)*,* and mild disease in *PKD2* mutations; patients without *PKD1* and *PKD2* mutation detected also have very mild disease^8–10^. Historically, management of ADPKD focused on supportive care including blood pressure control, sodium restriction, and hydration.

Tolvaptan is a selective, competitive vasopressin receptor 2 (V2) antagonist which has been shown to reduce cAMP levels, cellular proliferation and fluid secretion of cystic epithelia in preclinical studies^11,12^. The results from the TEMPO 3:4 and REPRISE trials ushered in a new era in ADPKD management^13,14^. The TEMPO 3:4 study demonstrated that tolvaptan treatment reduced kidney growth by nearly 50% and reduced rate of eGFR loss by approximately 26% in patients aged 18 to 50 and eGFR ≥ 60 mL/min over a three year period^13^. By contrast, the REPRISE trial confirmed a reduction in rate of eGFR loss with tolvaptan over one year in a cohort of patients with moderate to severe kidney impairment^14^. As a result, tolvaptan is now approved in many jurisdictions as the standard of care for patients at high-risk of progression of ESKD. However, tolvaptan treatment is expensive and associated with a number of side-effects, including acute liver toxicity, acute kidney injury, gout, and most commonly, polyuria due to its aquaretic effect from vasopressin inhibition^13,15,16^.

While the aforementioned observations were derived from clinical trials, little information exists regarding real-world use of tolvaptan in patients living with ADPKD. In this single-center study, we report our findings on the patterns of tolvaptan usage in a large cohort of patients with ADPKD and examined the main reasons why patients considered to be at high-risk for ESKD progression were not on this treatment.

## Methods

### Study population

**Figure 1:**
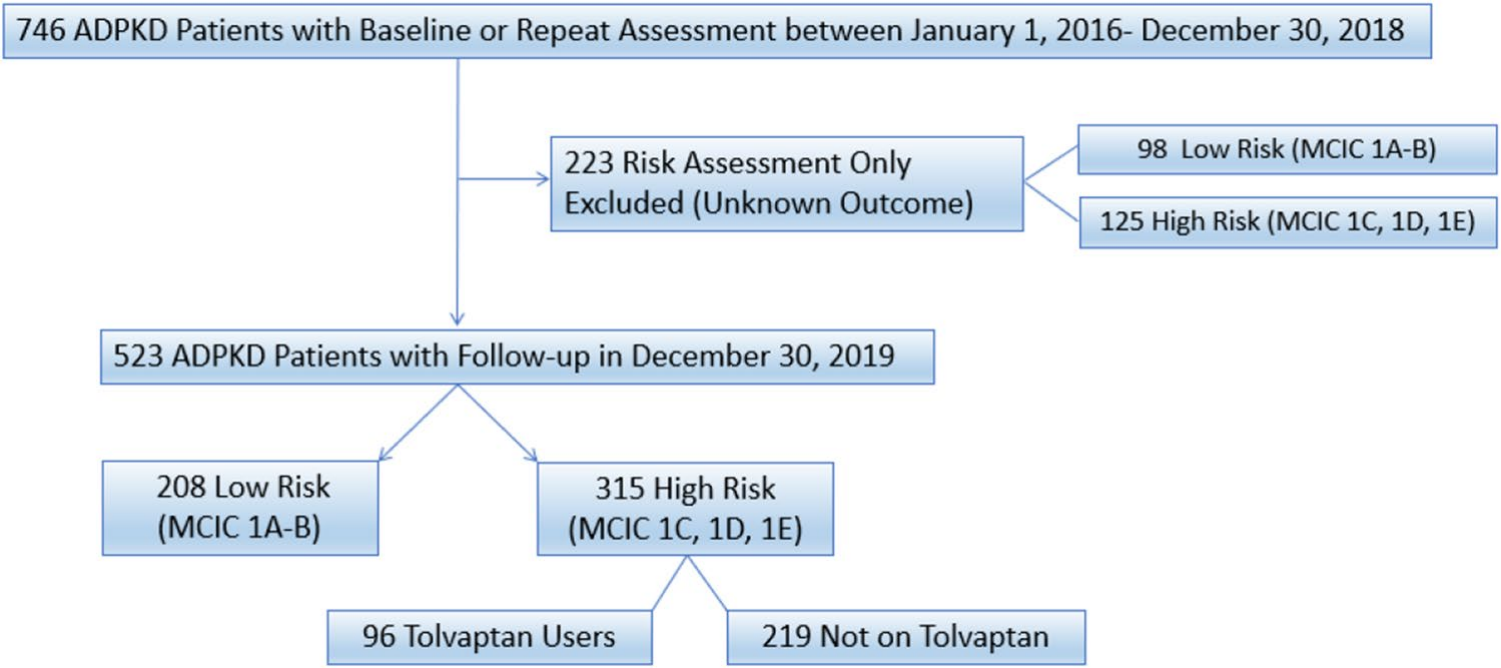
Flow diagram of study cohort. Of 746 current or new patients assessed at the Center for Innovative Management of Polycystic Kidney Disease between January 1, 2016–December 30, 2018, 233 were seen only once for risk assessment with management decisions provided by their primary nephrologists and were excluded given unknown outcome. The study cohort comprised of the remaining 523 patients followed primarily or co-managed with their primary nephrologists by the CIMPKD stratified by Mayo Clinic Imaging Classification and usage of Tolvaptan. Mayo Clinic Imaging Classification, MCIC.

The Center for Innovative Management of Polycystic Kidney Disease (the “Center” or “CIMPKD”; www.cimpkd.ca) is a PKD specialty center that provides risk assessment by MRI kidney imaging and clinical care to patients in the Greater Toronto Area (population of ~ 6.7 million, circa 2021). Between January 1, 2016 and December 31, 2018, 746 current or new patients were seen at the Center reflecting a referral experience from more than 100 academic and community nephrologists; 223 (30%) of patients who were referred by their community nephrologists for risk assessment and only seen once were excluded from this study since their subsequent tolvaptan therapeutic decisions were unknown (Fig. 1). The study cohort comprised 523 (70%) who were followed by the Center alone or co-managed with their referring nephrologists.

All 746 patients were aged 18 years or older, with confirmed ADPKD by standardized imaging criterion^17,18^; all had MRI-based kidney imaging and an assignment of Mayo Clinic Imaging Class (MCIC)^19–22^and most had *PKD1* and *PKD2* genetic screening^10^. Tolvaptan was proposed to all patients who met the criteria (MCIC 1C, 1D or 1E) for treatment with the drug. During their first visit, all patients eligible for tolvaptan treatment were asked if they had already been on the treatment. For those who were not on treatment we then proposed tolvaptan usage and the risks and benefits of the drug were explained: their decision regarding therapy was documented in the clinic chart including reason for refusal.

This study followed the STROBE Checklist for Observational Studies. The following clinical information was collected through retrospective chart review of the patients at the time of their MRI risk assessment: age, sex, serum creatinine, eGFR calculated with CKD-EPI, creatinine clearance (CrCl), CKD stage, genetic testing, baseline total kidney volume adjusted for height (ht-TKV) measured by MRI, and Mayo Clinic Imaging Class (MCIC). Patients with atypical (i.e. MCIC class 2) or non-ADPKD cystic kidney disease were excluded^21^. The prevalence of atypical ADPKD at our center is estimated to be approximately 9%^23^. Genetic testing was performed in a single research laboratory in Toronto using a validated long-range PCR protocol and bidirectional sequencing of the coding region and splice junctions of *PKD1* and *PKD2*. Nonsense, frameshift, and canonical splice-site mutations were classified as protein-truncating mutations. Nonsynonymous missense or atypical splice site mutations were grouped as non-truncating mutations^10^. All procedures were in accordance with the ethical standards of the University Health Network and with the 1964 Helsinki Declaration. All participants and/or their legal guardians provided signed informed consent according to a prespecified protocol approved by the Institutional Ethics Review Board at the University Health Network in Toronto.

### Statistical analysis

Descriptive statistics were performed using Graph Pad Prism 8.0 (GraphPad Software, La Jolla, CA.). Continuous variables were reported as mean (SD) or median (interquartile range) and categorical variables were described as number (%). Statistical testing for differences between groups for continuous variables was performed using Student’s t-test while categorical variables were compared using Chi-Square or Fisher's exact test as appropriate.

## Results

### Clinical characteristics

Figure 1 shows a flow diagram for the assembly of our study cohort. Our study cohort comprised 523 patients who were followed, or co-managed with their primary nephrologists, by CIMPKD. Compared to the excluded patients, our study cohort was younger (46 ± 14 vs. 51 ± 16 years), had a higher eGFR (79 ± 27 vs. 68 ± 35 ml/min/1.73 m^2^; *p* value < 0.05), and were less likely to have CKD stages 3–5 (25% vs. 42%; *p* value < 0.05). However, the height-adjusted total kidney volume (median: 680 vs. 670 ml/m) and percent distribution in MCIC between the two groups were not different (Table 1).

**Table 1:** Clinical characteristics of study patients and excluded patients.

Clinical features^a^	Full cohort (N = 746)	Study patients (n = 523)	Excluded patients (n = 223)
Patient number	746	523	223
Age, mean ± SD	49 ± 14.8	46 ± 14.2	51 ± 15.7
Male sex	357 (47.8%)	238 (45.5%)	119 (53.4%)
eGFR (CKD-EPI, ml/min/1.73 m^2^), mean ± SD	76 ± 29.8	79 ± 26.7	68 ± 35*
CrCl (ml/min), mean ± SD	90 ± 40.9	94 ± 35	81 ± 41*
CKD stage			
CKD 1/2	517 (69.3%)	389 (74.4%)	128 (57.4%)*
CKD 3	174 (23.3%)	120 (22.9%)	54 (24.2%)*
CKD 4/5	55 (7.4%)	14 (2.7%)	41 (18.4%)*
Genetic mutation			
PKD1 PT	203 (27.2%)	143 (27.3%)	60 (26.9%)
PKD1 NT	176 (23.6%)	124 (23.7%)	52 (23.3%)
PKD2	172 (23.1%)	136 (26%)	36 (16.1%)
No mutation	147 (19.7%)	103 (19.7%)	44 (19.7%)
Not available	48 (6.4%)	17 (3.2%)	31 (13.9%)*
ht-TKV, median (IQR)	682 (382–1149)	684 (380–1144)	672 (382–1146)
Mayo Clinic Imaging Classification			
1A/1B	306 (41.0%)	208 (39.8%)	98 (44%)
1C	226 (30.3%)	168 (32.1%)	58 (26%)
1D/E	214 (28.7%)	147 (28.1%)	67 (30%)

PKD1, PT PKD1 protein truncating mutation; PKD1 NT, PKD1 non-truncating variants (i.e. indel small in-frame deletion/insertion, nonsynonymous missense, or atypical splice site variants); PKD2, PKD2 mutation.

^a^Data are presented as the number (percentage) of patients, unless otherwise reported.

*P < 0.05 for pairwise comparisons to the reference.

Among the study cohort, 315 of 523 (60%) patients were calculated to be high-risk by MCIC (1C-1E); however, only 96 of 315 (30%) of these high-risk patients were treated with tolvaptan at their respective last follow-ups. Among the 219 ADPKD patients not on tolvaptan at last follow-up, less than 2% were previously treated with tolvaptan. Comparing the high-risk patients treated versus not treated with tolvaptan, the untreated patients were younger (43 ± 14 vs. 46 ± 13 years), had a higher eGFR (82 ± 26 vs. 61 ± 27 ml/min/1.73 m^2^; *p* value < 0.05), a lower height-adjusted total kidney volume (median: 848 vs. 1329 ml/m; *p* value < 0.05), and were more likely to have CKD stages 1–2 (79% vs. 41%; *p* value < 0.05) and MCIC 1C (63% vs. 31%; *p* value < 0.05) (Table 2).

**Table 2:** Clinical characteristics of patients with Mayo Clinic Imaging Class 1C, 1D, or 1E on tolvaptan versus not on tolvaptan at last follow-up (n = 315).

Clinical features^a^	Tolvaptan	Not on tolvaptan
Patient number	96	219
Male sex	55 (57.3%)	102 (46.6%)
Age, mean ± SD	46 ± 13	43 ± 14
eGFR (CKD-EPI, ml/min/1.73 m^2^), mean ± SD	61 ± 27	82 ± 26*
CrCl (ml/min), mean ± SD*	76 ± 33	96 ± 6*
CKD stage		
CKD 1/2	39 (40.6%)	173 (79%)*
CKD 3	49 (51%)	42 (19.2%)*
CKD 4/5	8 (8.3%)	4 (1.8%)*
Genetic mutation—no. (%)		
* PKD1* PT	43 (44.8%)	73 (33.3%)
* PKD1* NT	27 (28.1%)	56 (25.6%)
* PKD2*	17 (17.7%)	55 (25.1%)
No *PKD1* and *PKD2* mutation detected	7 (7.3%)	30 (13.7%)
Not available	2 (2.1%)	5 (2.3%)
ht-TKV, median (IQR)*	1329 (949–1772)	848 (609–1298) *
Mayo Clinic Imaging Class*		
1C	30 (31.2%)	138 (63%)*
1D/1E	66 (68.8%)	81 (37%)*

*PKD1* PT, *PKD1* protein truncating mutation; *PKD1* NT, *PKD1* non-truncating mutations; *PKD2*, *PKD2* mutation.

^a^Data are presented as the number (percentage) of patients, unless otherwise reported.

* P < 0.05 for pairwise comparisons to the reference.

### Reasons for not considering treatment with Tolvaptan

**Figure 2:**
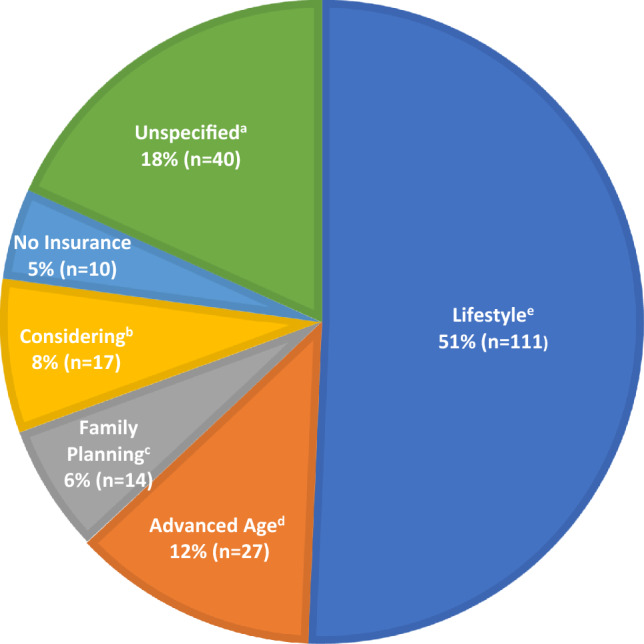
Reasons for not using tolvaptan among ADPKD patients with Mayo Clinic Imaging Classification 1C, 1D, 1E (n = 219). Patient were from the cohort followed primarily or co-managed with their primary nephrologists by the Center for Innovative Management of Polycystic Kidney Disease. ^a^Unspecified: No reason provided in the response. ^b^Considering: Patients who are considering tolvaptan but remain undecided. ^c^Family planning: Planning or contemplating pregnancy. ^d^Advanced age: Age ≥60 with perceived reduced benefit of tolvaptan usage. ^e^Lifestyle: Patients who had refused tolvaptan due to being unable to handle the possible aquaretic effect of the medication due to either their occupation or social circumstances.

**Figure 3:**
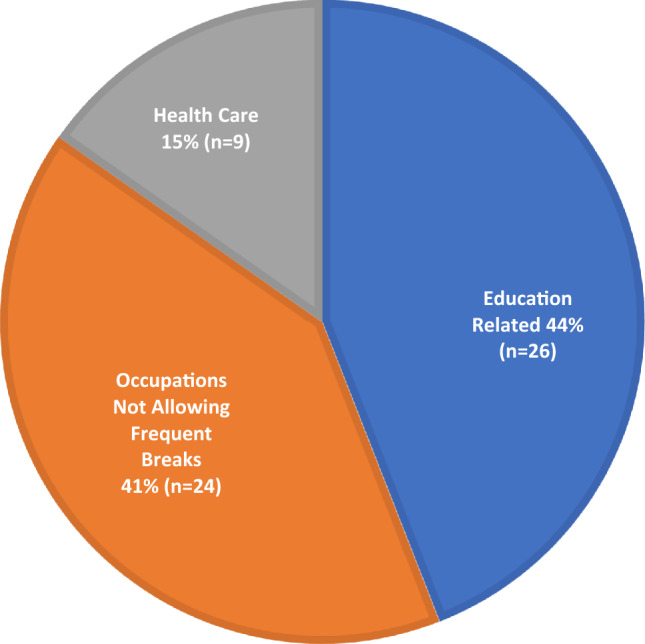
Occupations in patients with Mayo Clinic Imaging Class 1C, 1D, or 1E citing their profession as the lifestyle reason for not taking tolvaptan (n = 59).

We examined the reasons why patients with MCIC 1C, 1D, or 1E were not on tolvaptan therapy at their last follow-up (Fig. 2). The most common reasons cited for not considering tolvaptan treatment included the impact of aquaretic effect on lifestyle in 111/219 (51%), advanced age (age ≥ 60) in 27/219 (12%), planning for pregnancy in 14/219 (6%) and lack of private insurance coverage in 10/219 (5%). Among those not on tolvaptan due to lifestyle concerns, 59/111 (53%) cited their occupation as being incompatible with the aquaretic side-effect; they included those involved in education—students or teachers, health care providers, and other occupations not allowing frequent breaks including factory workers and bank tellers **(**Fig. 3**)**. Forty of 219 (18%) patients did not provide a specific reason for not considering tolvaptan therapy. Compared to those who provided a reason for their refusal to consider tolvaptan, they tended to be older (49 ± 8 vs. 42 ± 15 years), were more likely to belong to MCIC Class 1C (70% vs. 61%) and carry a *PKD2* mutation (40% vs. 22%) (Table 3).

**Table 3:** Clinical characteristics of patients with Mayo Clinic Imaging Class 1C,1D, or 1E not on tolvaptan for who provided versus did not provide a specific reason for declining tolvaptan therapy.

Clinical characteristics	Reason specified	Reason not specified
Patient number	179	40
Age	42 ± 14.9	49 ± 8.2*
Male sex	82 (45.8%)	20 (50%)
eGFR (ml/min/1.73 m^2^)	84 ± 25.6	70 ± 26.5*
CKD stage		
CKD 1/2	148 (82.7%)	25 (62.5%)*
CKD 3	29 (16.2%)	13 (32.5%)*
CKD 4/5	2 (1.1%)	2 (5%)
Mayo Clinic Imaging Class		
1C	110 (61.4%)	28 (70%)
1D-1E	69 (38.5%)	12 (30%)
Genetic mutation		
* PKD1* PT	68 (37.9%)	5 (12.5%)*
* PKD1* NT	49 (27.4%)	7 (17.5%)*
* PKD2*	39 (21.8%)	16 (40%)*
No *PKD1* and *PKD2* mutation	22 (12.3%)	8 (20%)*
Not available	1 (0.6%)	4 (10%)*

*PKD1* PT, *PKD1* protein truncating mutation; *PKD1* NT, *PKD1* non-truncating variants; *PKD2*, *PKD2* mutation.

* P < 0.05 for pairwise comparisons to the reference.

## Discussion

Tolvaptan is the first and only approved disease-modifying drug for treatment of ADPKD^13^. Thus, identifying high-risk patients with rapid progression based on MCIC who are most likely to benefit from therapy is an essential step of contemporary management in ADPKD^10^.

In this study we described a large cohort of patients with ADPKD and compared the clinical characteristics of those treated versus not treated with tolvaptan. Using MCIC, we found that 60% (315/523) of our study patients were considered to be at high-risk for progression to ESKD and yet only 30% (96/315) of them were treated with tolvaptan at their respective last follow-up. Of note, the benefit of tolvaptan treatment to delay ESKD is greatest among those with higher baseline eGFR at treatment initiation^24^. Specifically, a recent modelling study using the data from TEMPO 3:4 and REPRISE trials suggests an average delay of kidney replacement therapy by 7.3 years when tolvaptan was administered in those with eGFR > 90 ml/min/1.73 m^2^^14,24^.

We found that concern regarding the aquaretic effect was the single most important barrier for tolvaptan treatment among the high-risk patients; especially those who were engaged in education (students and teachers) or employed in health care (nurse and doctors), manufacturing (assembly line works), and banking (tellers) where frequent washroom breaks throughout the day are challenging and rendering tolvaptan therapy impractical without special accommodations. Recent preclinical studies have shown that metformin may be a vasopressin-independent activator of water transport in the rat inner kidney medulla^25^ and decreased polyuria by 50% in tolvaptan-treated rats^26^. While diuretic use was excluded in both TEMPO 3:4 and REPRISE trials, a recent study suggests that thiazide diuretics may be considered as a safe second-line anti-hypertensive in ADPKD^22^. Given that thiazide diuretic is an established treatment for nephrogenic diabetes insipidus, it may lower urine output from tolvaptan treatment^27^. In this regard, a recent double-blind, randomized, controlled, crossover trial of 13 tolvaptan-treated patients with ADPKD showed short-term treatment with metformin reduced urine output from 6.9 ± 1.4 to 5.4 ± 1.5 L/day. By contrast, short-term treatment with a thiazide diuretic in the same trial reduced urine output from 6.9 ± 1.4 to 5.1 ± 1.5 L/day^28^. Thus, add-on therapy such as metformin or a thiazide may provide a modest effect in countering the polyuria and improve the tolerability of tolvaptan treatment. Moreover, the Tolvaptan-Octreotide LAR Combination in ADPKD (TOOL) trial recently reported that the addition of octreotide-long acting release (LAR) on top of tolvaptan attenuated the aquaretic effect of tolvaptan in patients with ADPKD. During one month treatment, the 24-h median urine output in those treated with tolvaptan and placebo was 1193 mL higher than those treated with tolvaptan and octreotide-LAR^29^. On the other hand, it is unclear whether by raising the urinary osmolality this approach may blunt the therapeutic effect of tolvaptan.

Two other common reasons cited for not considering tolvaptan treatment among the high-risk patients include (i) older age ≥ 60 (12%) and (ii) pregnancy or family planning (6%). Tolvaptan treatment for ADPKD in Canada is currently not covered by the universal health care system but primarily by patient’s private health insurance. For older high-risk patients who declined tolvaptan, a commonly cited reason was a lack of private drug coverage due to retirement. On the other hand, pregnancy and family planning may be only a temporary barrier for considering tolvaptan in some patients. These findings from our real-life cohort highlight the crucial importance of considering patient lifestyle, quality of life, and occupation into the decision to initiate tolvaptan.

Our study has a number of strengths including a large cohort of patients referred by more than 100 academic and community nephrologists from a single geographic region. Furthermore, we had detailed clinical, imaging, and genetic information available on the study participants. This study is unique as it is the first to routinely identify patient-reported reasons for not taking tolvaptan and the findings reflect patient priorities which has rarely been considered in studies of ADPKD. By contrast, our work is limited by a lack of longitudinal outcome data related to tolvaptan treatment tolerability. While tolerability of aquaretic effect was the major barrier identified, the NICE guidance developed based upon advice from patient experts suggests that it is possible to adapt to the aquaretic effects of tolvaptan on thirst over time^30^. In addition, the tolvaptan treatment preference is unknown in 223 patients who were excluded due to their being seen one time only for risk assessment. These excluded patients were older with a lower eGFR and more advanced CKD stages and might have a higher tolvaptan treatment preference than reported in the study cohort. Lastly, in-depth analysis of reasons for non-treatment would provide more insights into the nuances behind individual patient decisions such as health literacy, socio-economic status, concerns for privacy, discrimination, and job security, beyond simply the risks and benefits of the treatment in slowing disease progression. However, such a study may be best conducted using a focus group qualitative analytical approach.

In conclusion, we found that ~ 60% of a real-life cohort with ADPKD were considered to be at high risk for progression to ESKD but only 30% of these high-risk patients were treated with tolvaptan; most untreated high-risk patients had early stages of CKD with well-preserved eGFR and as such, were prime targets for tolvaptan therapy to slow disease progression. Our study highlights the importance of patient lifestyle including occupation and quality of life as a critical determinant to implementing tolvaptan therapy even in those at high-risk for disease progression. While tolvaptan is a disease-modifying therapy that has revolutionized treatment of ADPKD, in practice, the ability to tolerate aquaretic effect remains a significant under-recognized barrier, and strategies for mitigating this side-effect will likely improve patient endorsement to consider this treatment.

## Data Availability

Portions of the de-identified data are available upon reasonable request to the corresponding author, Y.P.
